# Relationship between carotid-femoral pulse wave velocity and decreased left ventricular diastolic function in patient with coronary heart disease: a cross-sectional study

**DOI:** 10.3389/fcvm.2025.1575077

**Published:** 2025-08-22

**Authors:** Haojie Gu, Guoliang Liang, Wenhao Zhang, Xinxin Gu, Qiong Zhang, Jiangwei Ma

**Affiliations:** ^1^ Intensive Care Unit, Fudan University Shanghai Cancer Center, Shanghai, China; ^2^ Department of Cardiovascular Medicine, Fengxian Central Hospital, Shanghai, China; ^3^ The Third School of Clinical Medicine, Southern Medical University, Guangzhou, Guangdong, China

**Keywords:** carotid-femoral pulse wave velocity, arterial elasticity, diastolic heart failure, coronary artery atherosclerotic heart disease, left ventricular isovolumic relaxation time constant T

## Abstract

**Background:**

Arterial compliance is an independent predictor of diastolic dysfunction. Invasive catheterization can accurately reflect diastolic function. However, studies on the invasive assessment of diastolic function are currently limited. This study aimed to determine whether the diastolic function of the heart can be predicted by combining noninvasive detection of arterial elasticity indicators.

**Methods:**

This cross-sectional study included 390 hospitalized patients suspected of having coronary heart disease and underwent coronary angiography (CAG) at the South Hospital of the Sixth People's Hospital Affiliated to Shanghai Jiaotong University from June 2020 to June 2021. According to the degree of coronary artery stenosis, they were divided into group A (control, *n* = 73), group B (coronary stenosis < 50%; *n* = 128), and group C (coronary stenosis ≥ 50%; *n* = 189). Data of all enrolled patients, such as clinical information as well as noninvasive ultrasound and invasive cardiac catheterization results, were registered.

**Results:**

Significant differences in Left ventricular ejection fraction (LVEF), Ratio of peak mitral valve blood flow during early diastolic period to mitral ring velocity (E/e'), Left atrial volume index (LAVI), Deceleration time (DT), Carotid-femoral pulse wave velocity (cfPWV), Maximum velocity of left ventricular rise (LV + dp/dtmax), Maximum velocity of left ventricular descent (LV-dp/dtmax) and the left ventricular isovolumic relaxation time constant T were observed among the three groups (*P* < 0.01). Bivariate correlation analysis showed that cfPWV was positively correlated with T (*r* = 0.69, *P* < 0.01), E/e'(*r* = 0.59, *P* < 0.01), LAVI (*r* = 0.47, *P* < 0.01). Multiple linear regression analysis showed that cfPWV was correlated with T (*r* = 0.44, *P* < 0.01), E/e' (*r* = 0.24, *P* < 0.01), LAVI (*r* = 0.17, *P* < 0.01). Binary Logistic regression analysis showed that elevated cfPWV was associated with an increased risk of left ventricular hypodiastolic function [odds ratio (OR) = 3.30, 95% confidence interval (CI): 2.18–4.98, *P* < 0.01]. ROC curve analysis showed that the area under the curve of cfPWV response to diastolic dysfunction was 0.88 (95% CI: 0.85–0.91).

**Conclusions:**

cfPWV is significantly associated with left ventricular diastolic dysfunction in patients with coronary heart disease, and may also be used as a sensitive indicator to predict left ventricular diastolic dysfunction.

## Introduction

Over the last 10 years, the incidence and mortality of diastolic heart failure (HF) have continued to significantly increase (1, 2). According to statistics, diastolic HF accounts for about half of the total number of HF patients (3–5) and coronary artery atherosclerotic heart disease (CHD) has become one of the leading causes (6). Patients with diastolic HF may be asymptomatic or have only mild symptoms at the early age of the disease, making it difficult to identify them (7). Meanwhile, timely diagnosis and effective treatment of diastolic HF remain challenging in clinical practice due to the involvement of various etiologies and/or the coexistence of multiple underlying diseases (8).

Arterial elasticity, as an indicator of arterial wall relaxation, is an independent predictor of cardiovascular morbidity and mortality(). In a group of hypertensive patients, Mottram et al. found that arterial compliance was an independent predictor of diastolic dysfunction (3). An American study found that increased arterial stiffness was associated with the occurrence and poor prognosis of HF, and HF patients already had abnormal arterial stiffness in the early stage (9). A decrease in arterial compliance is significantly associated with the prevalence of cardiovascular disease (CVD), and the detection of arterial elasticity is imperative for CVD prevention (10).

To determine arterial stiffness and elasticity, clinically accurate invasive testing has limited applicability due to several reasons such as the possibility of invasive damage and being inconvenient; thus, noninvasive testing is more widely used. Cervical and femoral pulse wave conduction velocity (cfPWV) can be used as the “gold standard” for noninvasive detection (11). In this regard, Huybrechts et al. reported that cfPWV was positively correlated with the degree of coronary artery lesions, and the vascular compliance decreased with the aggravation of coronary artery stenosis (12). Interestingly, for every 1-m/s increase in cfPWV, the relative risk of total cardiovascular events increased by 1.14 [95% confidence interval (CI): 1.09–1.20] times (13).

However, previous studies have all used Ratio of peak mitral valve blood flow during early diastolic period to mitral ring velocity (E/e') or Ratio of peak e to peak a in the forward mitral valve flow (E/A) value to judge cardiac diastolic dysfunction (14, 15). As invasive examination the left ventricular isovolumic relaxation time constant T (A constant, calculated as described in the Methods section) is relatively independent of other parameters, it can be more accurately and regarded as the “gold standard” for evaluating cardiac diastolic function (16). While non-invasive techniques represent the primary clinical approach for evaluating diastolic function, invasive metrics such as the left ventricular isovolumic relaxation time constant T offer indispensable value in scenarios demanding utmost precision, direct measurement, resolution of complex diagnostic dilemmas, intraoperative real-time monitoring, or when serving as the research gold standard for validating novel methodologies. The application of these invasive indicators is predominantly concentrated within cardiac catheterization laboratories, cardiac operating rooms, heart transplant programs, and dedicated clinical and basic science research investigations. Therefore, based on previous literature, we hypothesized there could be a potential correlation between noninvasively measured cfPWV and early diastolic dysfunction. We explored the potential relationship between changes in arterial compliance and the occurrence of coronary heart disease and early cardiac function changes could be revealed through the determination of non-invasive arterial elasticity indexes combined with the assessment of invasive cardiac diastolic function, so as to prospectively predict the development of coronary artery disease and heart failure in patients.

## Methods

### Study design, setting, duration, and patients

In this cross-sectional study, we collected clinical information on 390 patients and recorded relevant indices reflecting the patients' arterial elasticity and left ventricular diastolic function at the time they underwent noninvasive echocardiography and coronary angiography (CAG).

This study included 390 hospitalized patients (188 men and 202 women) suspected of having coronary heart disease and underwent CAG at the South Hospital of the Sixth People's Hospital Affiliated to Shanghai Jiao Tong University from June 2020 to June 2021. All patients provided written informed consent (Approved by the Ethics Committee of the Sixth People's Hospital Affiliated to Shanghai Jiao Tong University, full name: the Shanghai Fengxian District Central Hospital Medical Ethics Committee, reference number: 2014-KY-06).

CAG was performed after admission, and the patients were divided into three groups according to the degree of coronary artery stenosis: group A (control, without coronary artery stenosis, *n* = 73), group B (coronary stenosis <50%; *n* = 128); and group C (coronary stenosis ≥ 50%; *n* = 189).

### Inclusion and exclusion criteria

The inclusion criteria were as follows: (1) having diagnostic criteria for angina pectoris based on the Guideline on the assessment and management of cardiovascular risk in China 2019 (17); (2) complete availability to clinical data; (3) age between 20 and 75 years old.

The exclusion criteria were: (1) having HF grades Ⅲ or Ⅳ based on the New York Heart Association (NYHA) classification (18); (2) having cardiac ultrasonography indicating various organic heart diseases, such as cardiomyopathy, valvular heart disease, ventricular hypertrophy, myocardial amyloidosis, congenital heart disease, ventricular aneurysm, and pericardial disease; (3) having measured the cardiac function under color Doppler cardiac ultrasound, and left ventricular ejection fraction (LVEF) < 50%; (4) having acute or old myocardial infarction; (5) the presence of certain underlying diseases including chronic obstructive pulmonary disease, arrhythmia, renal insufficiency; and (6) positive history of iodine allergy.

### Data collection

Demographic and some clinical data of patients were collected retrospectively, including sex, age, history of hypertension, diabetes, and smoking. Hypertension was defined as systolic blood pressure ≥140 mmHg and/or diastolic blood pressure ≥90 mmHg or self-reported hypertension and use of anti-hypertensive medication. Diabetes was defined as fasting blood glucose ≥7.0 mmol/L or Glycosylated Hemoglobin (HbAlc) ≥ 6.5% or self-reported diabetes history or receiving hypoglycemic therapy. Patients were divided into smokers (those with a self-reported smoking history), nonsmokers (those who have never smoked). Height and weight were measured upon admission, and Body surface area (BSA) was calculated using the international formula: BSA (m^2^) = 0.0061 × height (cm) + 0.0124 × weight (kg) −0.009. Continuous numeric variable, such as fasting venous blood glucose, blood lipids (including triglycerides, total cholesterol, high density lipoprotein cholesterol, and low-density lipoprotein cholesterol), serum creatinine, troponin I (cTNI), and B-type natriuretic titanium (BNP) levels of all patients were also collected. According to the American Heart Association and based on the Gensini score (19), coronary stenosis was classified as ≤25%; ≤26%−50%; ≤51%−75%; ≤76%−90%; ≤91%−99%; and complete occlusion. The final Gensini score was obtained by multiplying the stenosis score of the evaluated artery by the correlation coefficient.

### Preparations for procedures

Within 24 h before the procedure, the patient was instructed to stop taking vasoactive drugs, avoid strenuous activities, and protect their emotional stability. Consumption of strong tea, coffee, tobacco, and alcohol were forbidden within 6 h, and the room temperature was kept at 22°C−26°C. The patient was placed in a supine position and allowed to quietly rest for 30 min and then connected to the electrocardiogram (ECG) machine. The right brachial artery blood pressure was continuously measured three times, the average blood pressure was calculated, and the digital management system (e-DMS) was input. The mean arterial pressure was calculated as follows: [(mean systolic pressure + 2 × mean diastolic pressure)/3)] mmHg (1 mmHg = 0.133 kPa).

### Echocardiography

PHILIPS EPIQ 7C was used as an ultrasonic diagnostic instrument with a probe frequency of 2.5–3.5 MHz. The patients were placed in the left lateral position, and the parasternal long axis section of the left ventricle, the short axis section of the left ventricle, and the four and two ventricle section of the apex of the heart were taken. DT, E/e' were measured using pulsed-wave Doppler ultrasound and tissue Doppler imaging (TDI) techniques, left atrial volume (LAV) was measured using the Simpson biplane method, the atrial apex four-chamber section and left atrial two-chamber section were removed, the endocardial boundary was delineated during atrial diastole and LAV was measured, and LAV index (LAVI) was normalized by BSA. By considering the same sinus cardiac cycle that interferes with prelife systole, left ventricular end-diastolic volume (LVEDV), and left ventricular end- diastolic volume (LVESV) were measured, respectively. The value of LVEF was calculated. An experienced cardiac sonographer performed the above procedure. The measurements were taken three times and then averaged.

Peripheral arterial ultrasound: PHILLPS IU22 color ultrasound instrument was used for examination, and digital management system (e-DMS) was randomly configured. All the patients were decubed, a cfPWV pressure receptor was placed at the most obvious position of carotid artery and femoral artery pulsation on the right side, and the body surface distance between the two points was measured and input into the computer. The shape of the pulse wave was analyzed, and the cfPWV was automatically calculated according to the distance between the two pulse waves compared with the pulse wave conduction time. A deputy director or the above sonographer independently completed the aforementioned operations, and two professional deputy chief physicians performed the image measurement. If necessary, the measurement could be repeated three times and the average value taken.

### Coronary angiography

A large C-wall digital subtraction x-ray system from Siemens (model: AXIOM Artis Zee Celling, serial number: 147191, identification number: 720-939180) and a matching multichannel physiograph were used for hemodynamic determination and cardiac catheters. CAG was performed on the right radial artery of the patient as the site of puncture needle, routine disinfection was carried out, sterile towel was laid on, sterile gloves were worn, and local infiltration anesthesia was performed with 1% lidocaine. After successful puncture of the radial artery by Seldinger method with the most obvious radial artery pulsation, the 6 F radial artery sheath was placed along the ultra-smooth guide wire, and the bubbles in the sheath were fully extracted. Approximately 5 ml of 1% lidocaine injection, 0.3 mg of nitroglycerin injection, and 3,000 IU of heparin were injected. The optimal imaging position was chosen, and a 5 F multipurpose angiography catheter was used for imaging. Use approximately 50–150 ml of iopamidol and select an appropriate catheter and guidewire [commonly Judkins catheter, 0.035-inch (0.89 mm) J-tip guidewire] to perform coronary angiography. CAG examination was performed by experienced cardiologists. Multi-individual site imaging was also performed. Modified Gensini scoring criteria (20) were adopted for Gensini scoring.

### Left ventricular angiography

**Figure 1:**
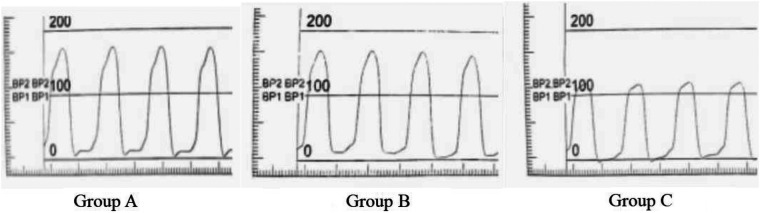
Comparison of pressure curves of each group.


In this study, the patient was photographed at the right anterior oblique position of 30° at a speed of 50 frames/s. A 6F pigtail catheter and a high-pressure syringe were used to rapidly inject iodixanol, a contrast agent. Furthermore, an x-ray machine was used for playback and measurement. The ECG and left ventricular pressure curves were synchronously recorded (at least five continuous cardiac cycles) and stored on a magnetic disc for offline analysis. Through the left ventricular pressure curve, the maximum velocity of left ventricular rise (LV + dp/dtmax), maximum velocity of left ventricular descent (LV-dp/dtmax) can be measured. The + dp/dtmax in systole, the -dp/dtmax in diastole, and the left ventricular isovolumic relaxation time constant T of the left ventricle were measured from the first-order differential curve of the left ventricular pressure curve. (T, normal value < 40 ms) (21) (Figure 1).

$$\begin{array}{ccc} T=-1/A & A=\mathrm{Ln}\Delta P/t & \Delta P=\mathrm{Pu}-\mathrm{Pl} \end{array}\;\;$$

(Pu, Pressure upper limit; Pl, Pressure lower limit; *t*, time of pressure changes between Pl and P2).

### Statistical analysis

The SPSS 25.0 statistical software was used for statistical analysis and GraphPad Prism 8.4.3 software was used for creating figures. The baseline demographic data and clinical variables were summarized. Continuous variables were expressed as mean ± standard deviation or median with interquartile range, whereas categorical variables were expressed as percentages and number of events. As applicable, analysis of variance for continuous variables and *χ*^2^ tests for categorical variables were used to compare if there were differences among the groups. Bivariate correlation analysis and stepwise linear regression analysis were employed to determine the correlation between cfPWV and the other variables. Meanwhile, binary logistic regression analysis was employed to explore the correlation between cfPWV, risk factors (age, sex, diabetes, blood glucose, hypertension, smoking history, blood lipid, creatinine, BNP, Gensini score), and diastolic dysfunction when left ventricular diastolic dysfunction defined as T ≥ 40. Furthermore, an ROC curve plotted with left ventricular isovolumic relaxation time constant (T ≥ 40) as the standard was created for the evaluation of diagnostic performance. The statistical significance of all the analyses was drawn at a two-sided significance level, which was 0.05.

## Results

**Figure 2:**
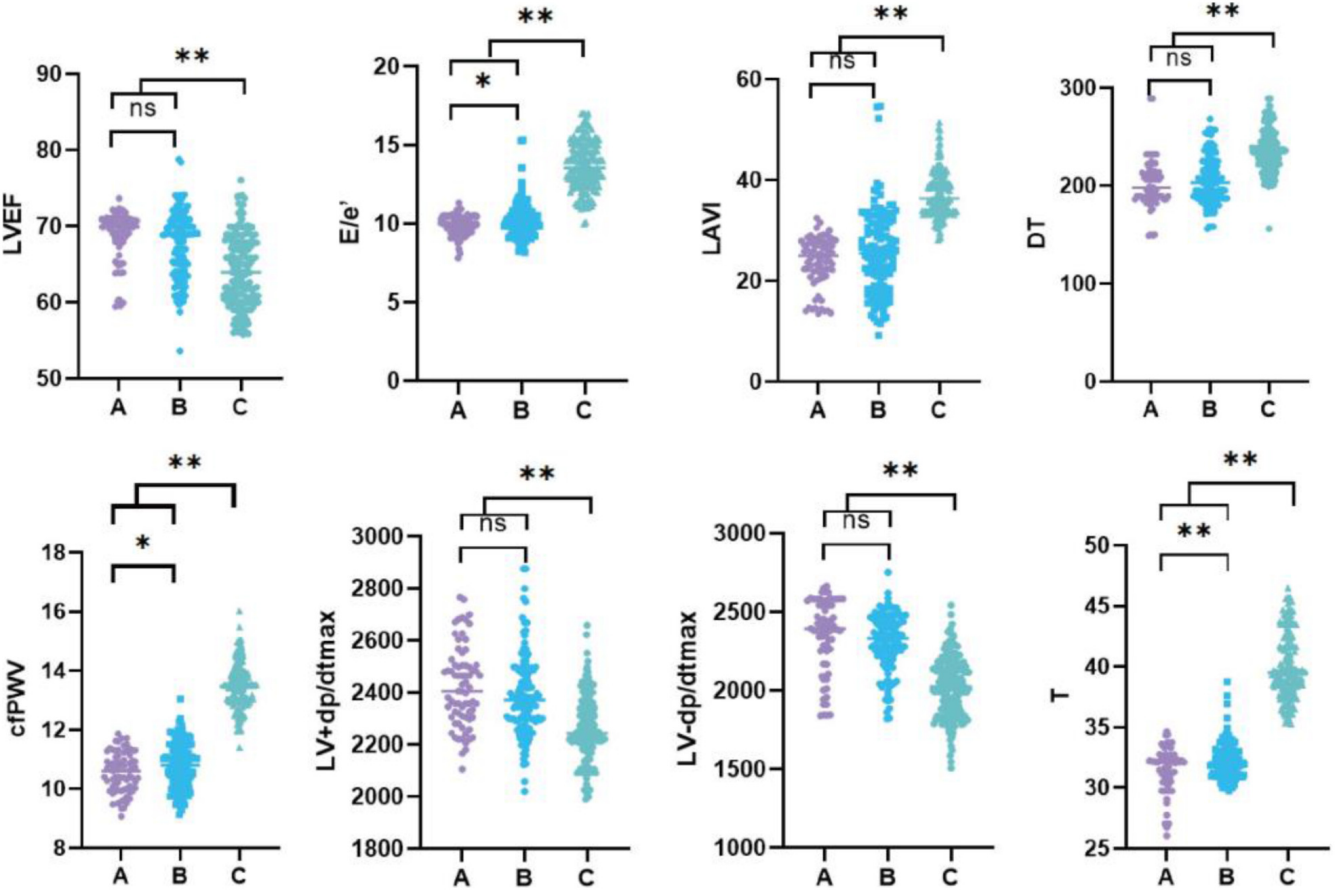
Statistical comparison of cardiac function indexes and cfPWV in each group. **(A)** Control group with no coronary stenosis; **(B)** coronary atherosclerosis group; **(C)** coronary heart disease group. EF, left ventricular ejection fraction (%); E/e’, ratio of mitral valve peak blood flow in early diastolic period to mitral ring velocity; LAVI, left atrial volume index; DT, deceleration time (ms); cfPWV, Carotid -femoral pulse wave conduction velocity; LV + dp/dtmax, maximum rate of left ventricular ascent; LV-dp/dtmax, maximum descending velocity of left ventricle; T, left ventricular isovolumic relaxation time constant. “*” was *p* < 0.05, “**” was *p* < 0.01.

The baseline data of the three groups were compared (Table 1; Figure 2). Our results indicated that the troponin I, LVEF, E/e', LAVI, DT, cfPWV, LV + dp/dtmax, LV-dp/dtmax, and T values as well as the Gensini score were statistically significant among the groups. In addition, based on pairwise comparison among the three groups, coronary artery stenosis worsened in different groups, significant differences were observed among the E/e', cfPWV, and T values.

**Table 1:** Baseline characteristics of the study patients.

Characteristics	A (*n* = 73)	B (*n* = 128)	C (*n* = 189)	*P* value
Age, years	57.16 (12.93)	58.55 (11.38)	59.66 (10.54)	0.453
Male (%)	40 (54.79)	59 (46.01)	89 (47.09)	0.261
Hypertension (%)	38 (52.05)	74 (57.81)	102 (53.97)	0.691
Diabetes (%)	15 (20.54)	28 (21.88)	49 (25.93)	0.563
Smoker (%)	23 (31.50)	29 (22.66)	49 (25.93)	0.389
BSA, m^2^	1.83 (0.11)	1.81 (0.17)	1.83 (0.16)	0.294
Fasting glucose, mmol/L	6.17 (5.63-6.71)	5.71 (5.36–6.03)	6.25 (5.95–6.54)	0.056
Serum cholesterol, mmol/L	4.51 (4.33–4.69)	4.30 (4.11–4.48)	4.39 (4.24–4.57)	0.380
Serum triglyceride, mmol/L	1.42 (1.25–1.60)	1.38 (1.27–1.48)	1.50 (1.39–1.62)	0.313
HDL, mmol/L	1.58 (1.39–1.77)	1.42 (1.30–1.54)	1.47 (1.37–1.55)	0.261
LDL, mmol/L	2.77 (2.63–2.92)	2.71 (2.59–2.82)	2.87 (2.73–3.01)	0.196
Serum creatinine, mmol/L	72.96 (62.89–83.03)	66.65 (63.86–69.44)	68.80 (66.57–71.04)	0.186
BNP, ng/ml	29.74 (17.02–42.46)	45.01 (33.96–56.06)	48.37 (38.17–58.56)*	0.118
cTNI (ng/ml)	0.01 (0.01–0.02)	0.02 (0.01–0.02)	0.03 (0.02–0.04)^**Δ^	0.004
Gensini score	0	5.36 (4.84–5.87)**	29.13 (24.80–33.46)^**ΔΔ^	<0.01
LVEF (%)	68.68 (3.37)	67.55 (4.39)*	64.21 (4.62)^**ΔΔ^	<0.01
E/e'	9.87 (0.64)	10.18 (1.12)*	13.67 (1.55)^**ΔΔ^	<0.01
LAVI (ml/m^2^)	23.73 (4.93)	25.67 (8.34)	37.24 (4.49)^**ΔΔ^	<0.01
DT (ms)	201.64 (24.53)	205.35（24.79）	235.21 (22.24)^**ΔΔ^	<0.01
cfPWV (m/s)	10.56 (0.68)	10.79 (0.78)*	13.47 (0.78)^**ΔΔ^	<0.01
LV + dp/dt_max_ (mmHg/s)	2421.27 (153.37)	2383.0 (154.49)	2261.86 (127.67)^**ΔΔ^	<0.01
LV-dp/dt_max_ (mmHg/s)	2350.32 (229.47)	2308.84 (177.10)	2022.35 (189.51)^**ΔΔ^	<0.01
T	31.49 (1.90)	32.15 (1.49)**	39.99 (2.66)^**ΔΔ^	<0.01

A, control group with no coronary stenosis; B, coronary atherosclerosis group; C, coronary heart disease group. BSA, body surface area; LDL, low-density lipoprotein; HDL, high-density lipoprotein; BNP, brain natriuretic peptide; EF, left ventricular ejection fraction (%); E/e’, ratio of mitral valve peak blood flow in early diastolic period to mitral ring velocity; LAVI, left atrial volume index; DT, deceleration time (ms); cfPWV, Carotid-femoral pulse wave conduction velocity; LV + dp/dtmax, maximum rate of left ventricular ascent; LV-dp/dtmax, maximum descending velocity of left ventricle; T, left ventricular isovolumic relaxation time constant; 1 mmHg = 0.133 kPa. Each subscript letter denotes a subset of categories whose row proportions do not difer signifcantly from each other at the 0.05 level. Values are mean ± standard deviation or *n* (%), *p* values were calculated using analysis of one-way ANOVA. Compared with control group “*” was *p* < 0.05, “**” was *p* < 0.01; Compared with coronary atherosclerosis group ^Δ^*p* < 0.05, ^ΔΔ^*p* < 0.01.

**Figure 3:**
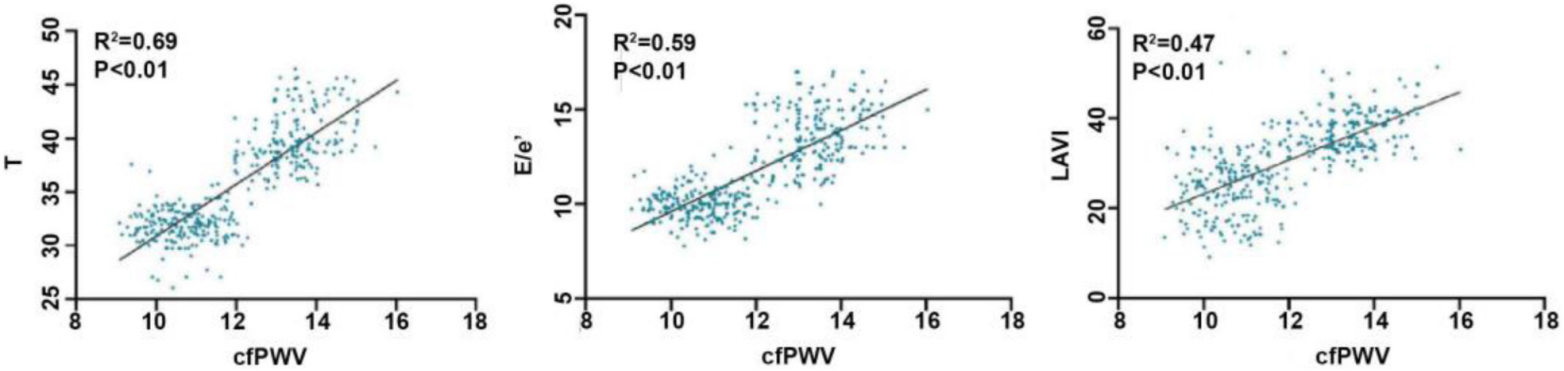
Correlation between cfPWV and T, E/e’, LAVI. cfPWV, carotid -femoral pulse wave conduction velocity; T, left ventricular isovolumic relaxation time constant; E/e’, ratio of mitral valve peak blood flow in early diastolic period to mitral ring velocity; LAVI, left atrial volume index.

The results of the bivariate correlation analysis indicated that cfPWV was positively correlated with E/e', LAVI, and T (all *P* < 0.01). But not with the other indicators with statistically significant differences between groups that may affect left ventricular diastolic function (LVEF, DT, LV + dp/dtmax, and LV-dp/dtmax). The results of the stepwise linear regression analysis indicated that cfPWV was significantly correlated with E/e', LAVI, and T (all *P* < 0.01) (Table 2; Figure 3).

**Table 2:** Bivariate correlation and linear regression analysis of cfPWV and clinical parameters.

Variables	*r*	*P* value
E/e'	0.59	<0.01
LAVI	0.47	<0.01
T	0.69	<0.01
Linear regression-method: stepwise *R* = 0.88		
T	0.44	<0.01
E/e'	0.24	<0.01
LAVI	0.17	<0.01
Constant	4.73	<0.01

cfPWV, Carotid -femoral pulse wave conduction velocity; T, left ventricular isovolumic relaxation time constant; E/e’, ratio of mitral valve peak blood flow in early diastolic period to mitral ring velocity; LAVI, left atrial volume index.

**Figure 4:**
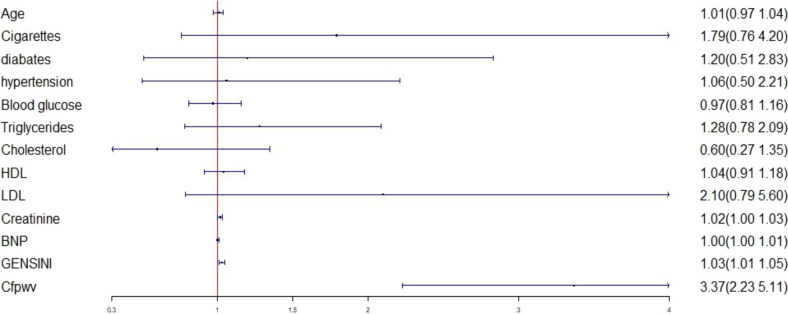
Logistic regression analysis between left ventricular diastolic dysfunction and various factors. LDL, low-density lipoprotein; HDL, high-density lipoprotein; BNP, brain natriuretic peptide; cfPWV, Carotid -femoral pulse wave conduction velocity.

Logistic regression analysis was conducted by considering left ventricular diastolic dysfunction (defined as T ≥ 40) as the dependent variable and sex, age, diabetes, hypertension, smoking history, blood lipid, cfPWV, blood glucose, creatinine, BNP, and Gensini score as independent variables. Logistic regression analysis revealed that elevated cfPWV was associated with an increased risk of left ventricular hypodiastolic function [odds ratio (OR) = 3.30, 95% confidence interval (CI): 2.18–4.98, *P* < 0.01] (Figure 4).

**Figure 5:**
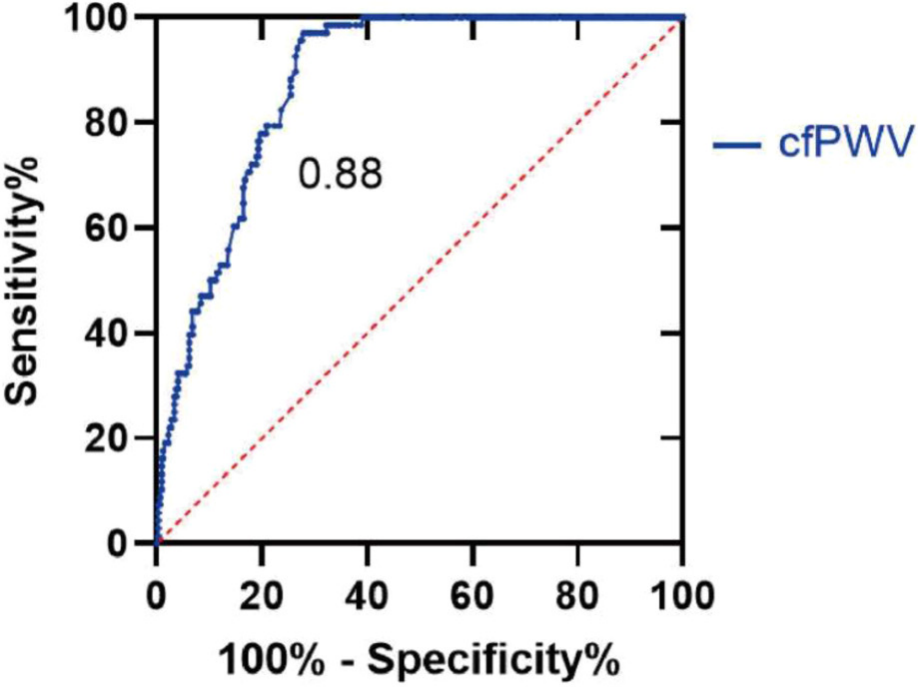
ROC curve. With T ≥ 40, the patients were divided into normal diastolic function group and reduced diastolic function group. T values in both groups were analyzed using ROC curves to determine the threshold for possible cfPWV prediction of diastolic dysfunction. cfPWV, Carotid -femoral pulse wave conduction velocity.

ROC curve analysis revealed that the area under the curve of cfPWV response to diastolic dysfunction was 0.88 (95% CI: 0.84–0.91), the standard error was 0.20, and the asymptotic significance was *P* < 0.01. The best threshold value for cfPWV to judge T was 12.91 m/s, with a sensitivity and a specificity of 0.97 and 0.72, respectively (Figure 5).

## Discussion

At present, there is a lack of effective treatment for diastolic HF. Thus, early detection of diastolic dysfunction and prevention of HF development are of great importance. Left ventricular diastolic dysfunction is also a predictor of CVD and mortality (22). Left ventricular relaxation rate represents the early stage of diastolic function, whereas left ventricular compliance represents the subsequent stage of diastolic function after left ventricular relaxation. Ultrasound ECG can detect early changes in left ventricular diastolic function through LAV measurements. According to the European Society of Cardiology (ESC) guidelines in 2016 and 2021, left atrial volume index (LAVI) > 34 ml/m^2^ is used as a reference index for diastolic dysfunction (23, 24). Therefore, E/e’ and LAVI are currently used as common indicators for the evaluation of left ventricular diastolic function via noninvasive echocardiography (23). Tsang et al. (25) reported that the specificity of LAVI > 34 ml/m^2^ for the detection of diastolic dysfunction was 100%. LAVI is closely associated with the severity and duration of left ventricular diastolic dysfunction and can be used as a reference index for diastolic function evaluation (26). Cardiac catheterization can be employed as the “gold standard” to evaluate cardiac diastolic function. The most commonly used index to evaluate isovolumic diastolic function is the diastolic time constant T, currently one of the most reliable methods for measuring diastolic function. According to a prospective, randomly selected community-based study, E/e' is moderately, but significantly, correlated with T (*r* = 0.53, *P* < 0.001) (11).

Arterial compliance, namely, arterial elasticity or arterial stiffness, is a functional vascular indicator that reflects the diastolic function and buffering capacity of the arterial wall. Arterial compliance tests include invasive and noninvasive methods. Invasive procedures include CAG, intra-arterial catheterization, or cardiac catheterization, whereas noninvasive detection methods include pulse pressure, pulse wave conduction velocity, reflection wave enhancement index, cuff pressure shock wave analysis, and high-resolution ultrasound. Recent studies have identified decreased arterial compliance as a strong predictor of the presence of atherosclerosis (5). This study found that as coronary artery disease worsened, the arterial elasticity index of the groups exhibited significant difference (*P* < 0.01), consistent with the results of previous studies (27).

It has been reported in literature that diastolic dysfunction is a sensitive indicator of early changes in cardiac function in patients with coronary heart disease, when cardiac systolic function is not changed, changes in left ventricular diastolic function can be found through echocardiographic measurements of the left atrial diameter and LAV (28, 29). Xanthopoulos et al. found that 68% of hospitalized patients who underwent CAG due to HFpEF had coronary heart disease (30). Furthermore, Abhayaratna found that increased arteriosclerosis was correlated with the severity of left ventricular diastolic dysfunction (31). In addition, Unagami et al. found that in patients with uremic hemodialysis, the higher the grade of diastolic dysfunction, the higher the score of peripheral atherosclerosis calcification (32). Left ventricular diastolic function is also an indicator of the prognosis of coronary heart disease. LVEDP ≥ 26.5 mmHg is one of the predictors of chronic HF and 1-year mortality (33). Ventricular arterial coupling is a key determinant of cardiovascular function. Clinically, patients with HF have abnormal arterial stiffness and ventricular vascular coupling (34). Increased arterial stiffness was associated with the occurrence and poor prognosis of HF and that HF patients already had abnormal arterial stiffness in the early stage (9). It is believed that atherosclerosis increases left ventricular afterload and reduces coronary perfusion. Therefore, early detection of diastolic dysfunction through the evaluation of arterial elasticity is of great importance for preventing the development of HF. The results of our previous study indicated that T was correlated with coronary Gensini score (35). This study found that with the aggravation of coronary atherosclerosis in the three groups, the contrast of E/e’ and T, reflecting left ventricular diastolic function, was significantly increased in each group, consistent with the results of previous studies.

cfPWV is the “gold standard” for noninvasive arterial elasticity testing, reflecting the central artery pressure and degree of stiffness of the large artery. Increased aortic PWV can cause changes in left ventricular diastolic function (36). In stiff arteries, pulse waves propagate faster and reflected waves merge with incident waves earlier, thereby increasing and decreasing the aortic systolic and aortic diastolic pressures, respectively, leading to a significant increase in left ventricular afterload and impaired coronary artery filling. Huybrechts et al. reported that cfPWV was positively correlated with the degree of coronary artery lesions and that vascular compliance decreased with the aggravation of coronary artery stenosis (12). This study found that the arterial elasticity index increased as the coronary artery disease worsened and that the decrease in vascular compliance caused by coronary artery sclerosis further affected the diastolic function of the heart, which resulted in impaired diastolic function, consistent with the results of previous studies. In a large population-based cross-sectional study, Kim et al. found that increased arterial stiffness, as measured using brachial–ankle pulse wave velocity, was correlated with abnormal diastolic function parameters, consistent with the results of our study (37). Abhayaratna et al. found that in the absence of identifiable cardiovascular risk factors, cfPWV increased with age and still exhibited a significant increase at the age of 40 (38). Kim et al. reported that increased arterial stiffness plays a pivotal role in the development of HF with preserved ejection fraction as well as left ventricular diastolic dysfunction in elderly women (39). In a study involving young, middle-aged, and elderly subjects without hypertension or other known risk factors for atherosclerosis, aortic stiffness was independently important for left ventricular filling injury, excluding other confounding factors such as age (40). Previous studies reported that the relative risk of total cardiovascular events increased by 1 m/s with cfPWV was 1.14 (95% CI: 1.09–1.20), and the risk was increased by 14% (3). In the present study, the correlation between arterial elasticity and diastolic function indices was investigated, and the results of bivariate correlation analysis indicated that cfPWV was positively correlated with T (*r* = 0.42, *P* < 0.01), E/e' (*r* = 0.26, *P* < 0.01), and LAVI (*r* = 0.22, *P* < 0.01). Meanwhile, multiple linear regression analysis revealed that cfPWV was moderately correlated with E/e', LAVI, and T (*P* < 0.01); the results indicated that the increase in cfPWV was closely associated with the decline in left ventricular diastolic function, which exhibited statistical significance. Logistic regression analysis revealed that increased cfPWV increased the risk of left ventricular diastolic dysfunction (OR = 3.30, 95% CI: 2.18–4.98, *P* < 0.01). Further ROC curve analysis revealed that the area under the curve of cfPWV response to diastolic dysfunction was 0.88 (95% CI: 0.84–0.91) and that the best threshold for cfPWV to determine T was 12.91 m/s, with a sensitivity and specificity of 0.97 and 0.72, respectively. These results suggest that cfPWV is a sensitive index for predicting diastolic dysfunction.

This study has some limitations. First, we only addressed the correlation between cfPWV and cardiac diastolic function. The specific mechanism involved needs to be further investigated. Second, only the diastolic function indicators, namely, T, LV + dp/dtmax, and LV-dp/dtmax, were included in the study; the other diastolic function indicators, such as isovolumic diastolic time, left ventricular maximum filling rate, and left ventricular stiffness, were not statistically analyzed. Third, as a single-center retrospective study with a relatively small sample size, selection bias cannot be avoided. Therefore, the results of this study need to be further validated in a multicenter randomized prospective study.

## Conclusion

The present study found that cfPWV is significantly correlated with left ventricular diastolic dysfunction in patients with coronary heart disease. It is also a sensitive indicator for predicting left ventricular diastolic dysfunction. Noninvasive detection of cfPWV could predict changes in cardiac diastolic function in patients with coronary heart disease. Therefore, its application in the screening of early HF in patients with coronary heart disease has potential value.

## Data Availability

The raw data supporting the conclusions of this article will be made available by the authors, without undue reservation.
